# Coexistence of two accessory flexor pollicis longus heads or coexistence of two-headed flexor pollicis longus with an unrecognized anatomical structure?

**DOI:** 10.1007/s00276-021-02721-w

**Published:** 2021-03-03

**Authors:** Nicol Zielinska, Bartłomiej Szewczyk, R. Shane Tubbs, Łukasz Olewnik

**Affiliations:** 1 Department of Anatomical Dissection and Donation, Medical University of Lodz, Lodz, Poland; 2 Department of Neurosurgery, Tulane University School of Medicine, New Orleans, LA USA; 3 Department of Neurosurgery and Ochsner Neuroscience Institute, Ochsner Health System, New Orleans, LA USA; 4 Department of Anatomical Sciences, St. George’s University, West Indies, Grenada

**Keywords:** Flexor pollicis longus, Additional head, Unrecognized structure, Anterior interosseous syndrome, Evolution, Interosseous membrane

## Abstract

The flexor pollicis longus (FPL) is located in the anterior compartment of the forearm. It is morphologically variable in both point of origin and insertion. An additional head of the FPL can lead to anterior interosseous syndrome. This report presents a morphological variation of the FPL (additional head in proximal attachment and bifurcated tendinous insertion in distal attachment) and an unrecognized structure that has not so far been described in the literature. This structure originates in six heads (attached to the FPL or interosseous membrane) that merge together, and inserts on to the FPL. All the variations noted have clinical significance, ranging from potential nerve compression to prevention of tendon rupture.

## Introduction

The deep muscle group in the anterior compartment of the forearm comprises the flexor digitorum profundus (FDP), flexor pollicis longus (FPL), and pronator quadratus (PQ) [12]. The FPL is unipennate, its proximal attachment being located on the anterior surface of the radius and the interosseous membrane. Its distal attachment is represented by a long tendinous slip coursing deep to the transverse carpal ligament as an element of the carpal tunnel and inserting on to the distal phalanx of the thumb [10]. This muscle is innervated by the anterior interosseous nerve (AIN), which provides motor innervation to all muscles in the deep anterior forearm compartment; it is a branch of the median nerve formed by medial and lateral cords from the C5–C8 nerve roots [31]. The anterior interosseous artery (a branch of the ulnar artery) supplies blood to the FPL [10].

The FPL is a primary flexor of the thumb, allowing for flexion at the metacarpophalangeal and interphalangeal joints. It also has minor responsibility for radial wrist deviation (also known as radial flexion), which is the bending of the wrist towards the thumb or radius side [14].

The FPL is very morphologically variable, and an additional head in the proximal attachment is particularly common. The results of one study showed that this additional head was more often present (66.66%) than absent [9]. There are also cases in which the FPL is fused with another muscle, for example the flexor digitorum longus [17].

Although spontaneous intratendinous flexor tendon ruptures are common in the FDP, they also occur in the FPL, though less frequently. The probable major causes of this pathology are repetitive impact forces or an uninterrupted load on the tendon, but the etiology has not yet been officially confirmed [13]. Another pathology can result from an additional FPL head: compression of the interosseous nerve, which is located underneath this head. This can lead to an entrapment neuropathy known as anterior interosseous nerve syndrome (AIS) [3].

The present report describes a morphological variation of the FPL. A distinctive feature in the proximal attachment is an additional head originating on the medial epicondyle. The distal attachment is divided into two tendons inserted on to the distal phalanx of the thumb. The report also describes an unrecognized structure originating in six heads attached to the FPL or interosseous membrane and merging together, and inserting on to the FPL. Both structures have considerable clinical significance. To our knowledge, this is the first description of such a case.

## Methods

Dissection began with removal of the skin from the area of the forearm and hand it was performed by traditional techniques [24–26]. These areas were exposed by the subcutaneous tissue and the antebrachial fascia. The next step included visualizing the FPL. The morphology, together with the location of the origin and the insertion were evaluated. Morphometric and anthropometric measurements of the belly and tendon of the FPL muscle and unrecognized structure were performed. An electronic digital caliper was used for all measurements (Mitutoyo Corporation, Kawasaki-shi, Kanagawa, Japan). Each measurement was carried out twice with an accuracy of up to 0.1 mm.

## Case report

**Fig. 1:**
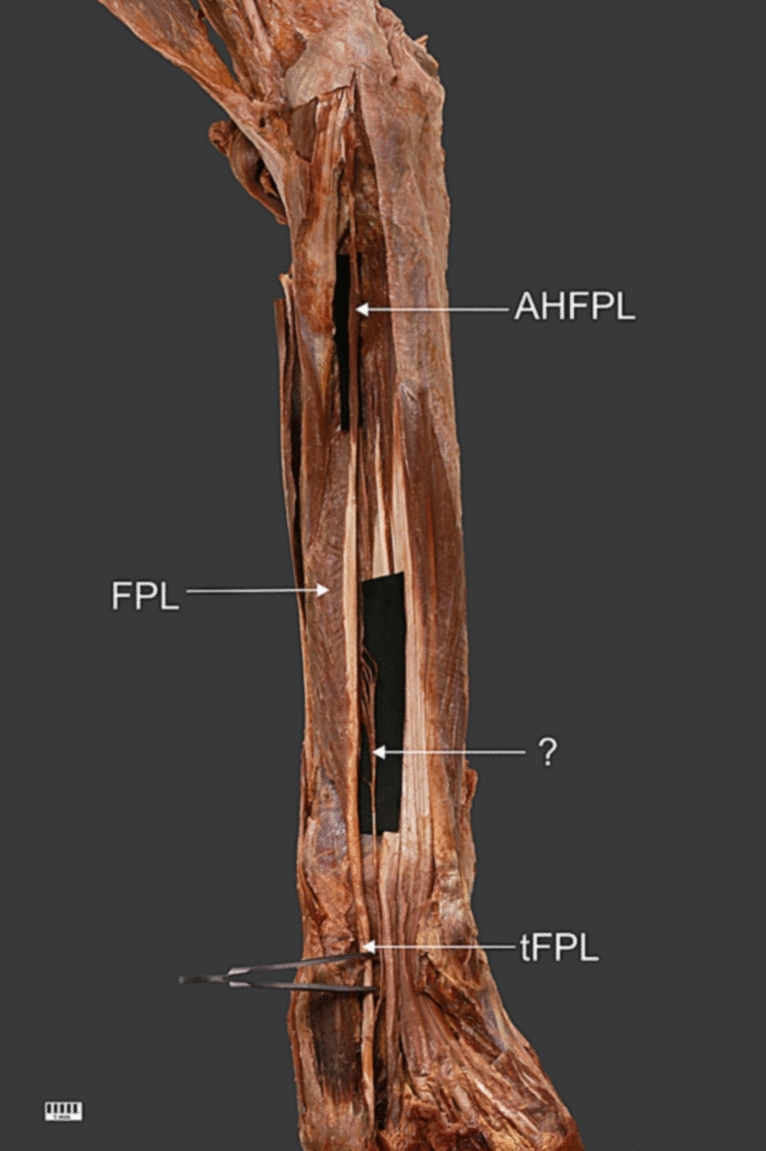
Total view of the right forearm. *AHFPL,* accessory head of the flexor pollicis longus; *FPL,* flexor pollicis longus; *?*, unrecognized anatomical structure; *tFPL,* tendon of the flexor pollicis longus

**Fig. 2:**
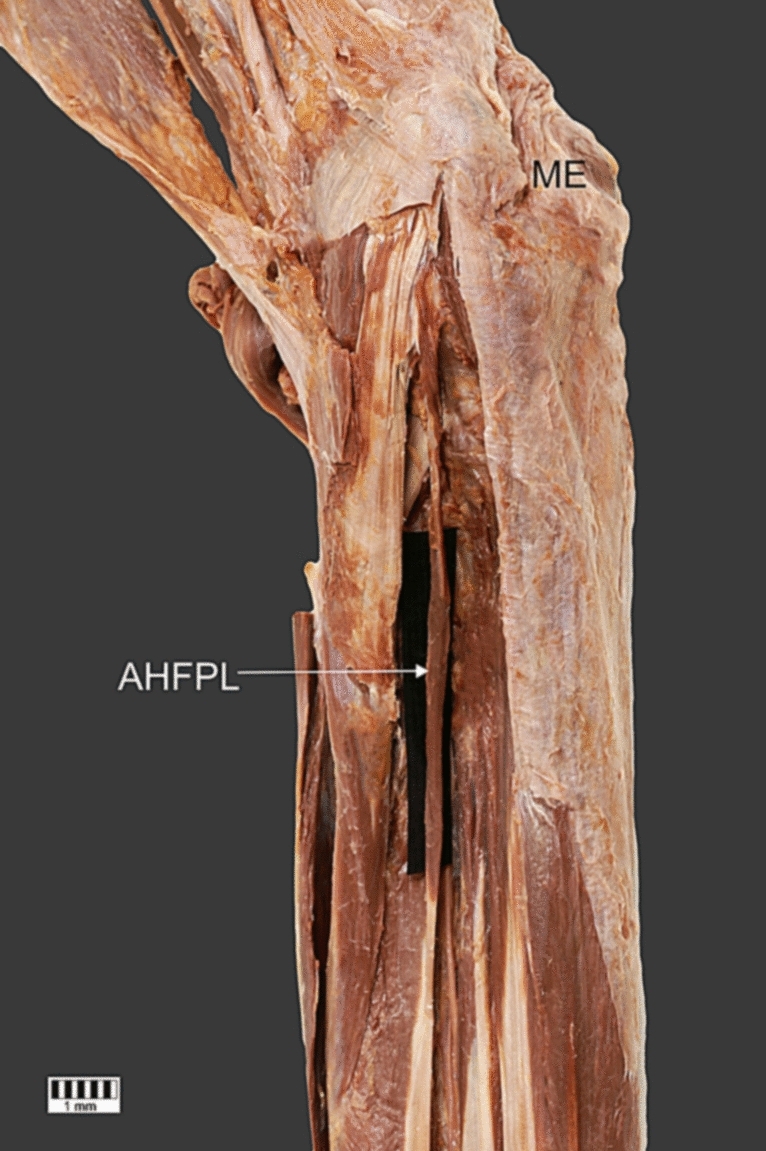
Proximal zoom of the accessory head of the flexor pollicis longus. Anterior view of the right forearm. *AHFPL,* accessory head of the flexor pollicis longus; *ME,* medial epicondyle

**Fig. 3:**
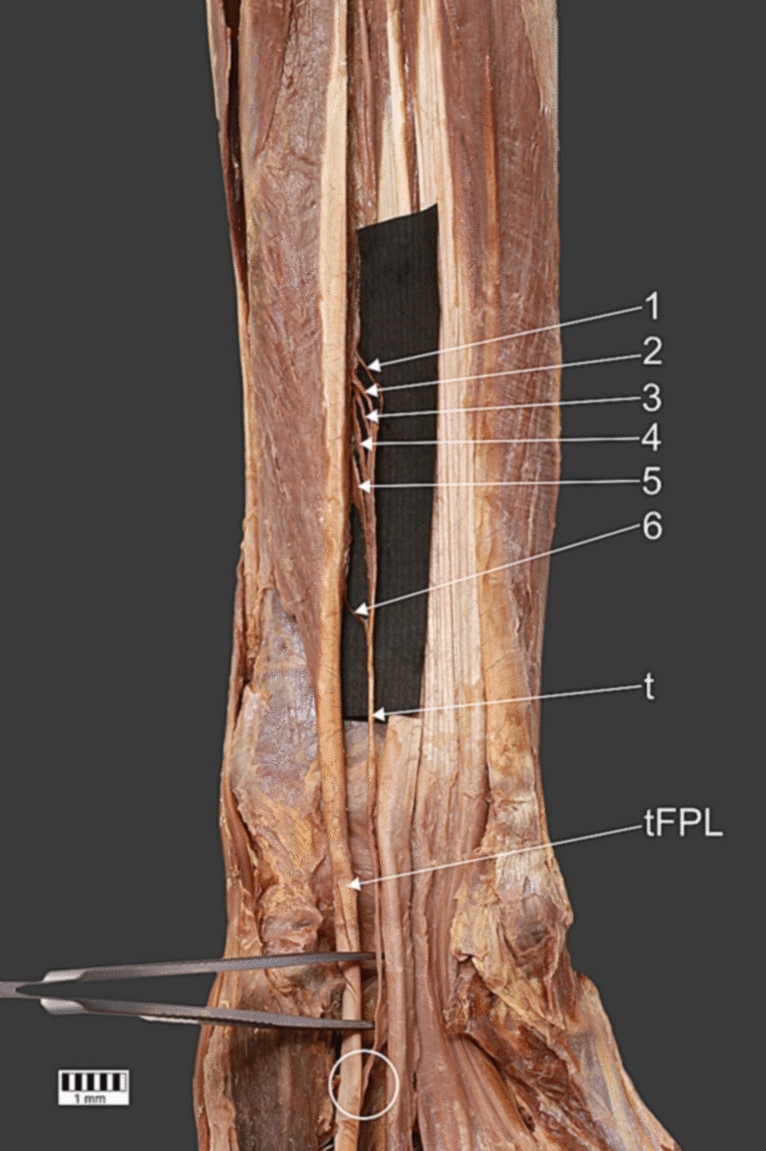
Distal zoom of the unrecognized anatomical structure. *1*, first head of the unrecognized anatomical structure; *2*, the second head of the unrecognized anatomical structure; *3*, the third head of the unrecognized anatomical structure; *4*, the fourth head of the unrecognized anatomical structure; *5*, the fifth head of the unrecognized anatomical structure; *6*, the sixth head of the unrecognized anatomical structure; *t*, tendon of the unrecognized anatomical structure tFPL tendon if the flexor pollicis longus muscle, *the white circle indicates the insertion/connection between the tendon of the* unrecognized anatomical structure and tendon of the flexor pollicis longus

**Fig. 4:**
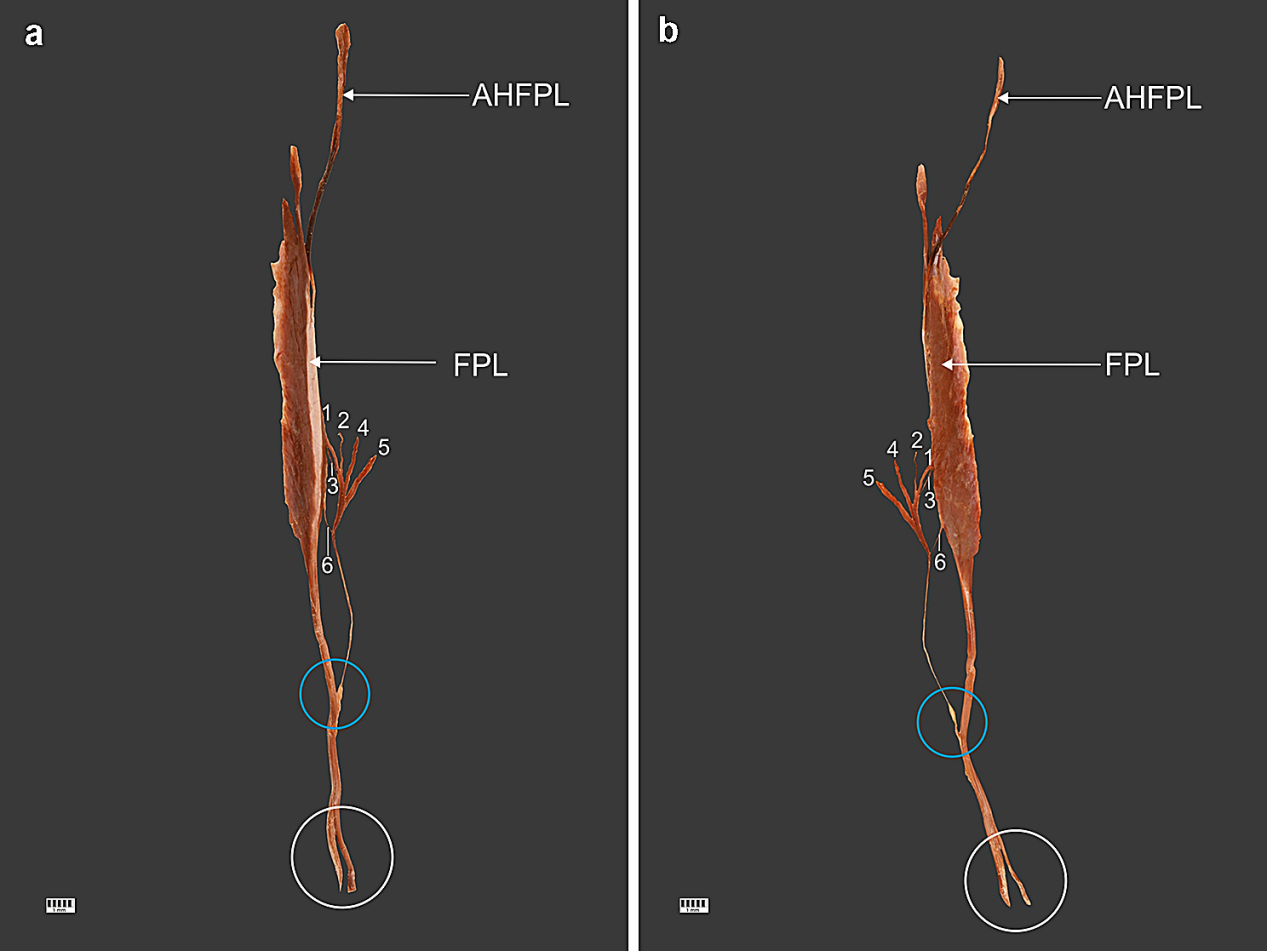
**a** Cut out flexor pollicis longus with accessory head of the flexor pollicis and the unrecognized anatomical structure. Ventral view. *AHFPL* accessory head of the flexor pollicis longus; *FPL* flexor pollicis longus muscle; *1* first head of the unrecognized anatomical structure; *2* the second head of the unrecognized anatomical structure; *3* the third head of the unrecognized anatomical structure; *4* the fourth head of the unrecognized anatomical structure; *5* the fifth head of the unrecognized anatomical structure; *6* the sixth head of the unrecognized anatomical structure; *the blue circle indicates connection between unrecognized anatomical structure with the tendon of the flexor pollicis longus*;* the white circle indicates the bifurcated of the tendon of the flexor pollicis longus.* **b** Cut out flexor pollicis longus with accessory head of the flexor pollicis and the unrecognized anatomical structure. Dorsal view. *AHFPL* accessory head of the flexor pollicis longus; *FPL* flexor pollicis longus muscle; *1* first head of the unrecognized anatomical structure; *2* the second head of the unrecognized anatomical structure; *3* the third head of the unrecognized anatomical structure; *4* the fourth head of the unrecognized anatomical structure; *5* the fifth head of the unrecognized anatomical structure; *6* the sixth head of the unrecognized anatomical structure; *the blue circle indicates connection between unrecognized anatomical structure with the tendon of the flexor pollicis longus*;* the white circle indicates the bifurcated of the tendon of the flexor pollicis longus*

A 69-year-old female cadaver was subjected to routine anatomical dissection for research and teaching purposes at the Department of Anatomical Dissection and Donation, Medical University of Lodz, Poland. Traditional dissection of the right upper limb [20–22, 24–26, 33, 34] revealed a morphological variant of the FPL and an unrecognized structure (Figs. 1, 2, 3, 4). The next stage of the investigation involved a detailed assessment of these two structures.

The origin consisted of two heads, one standard and one additional. The proximal attachment of the standard head was located on the anterior surface of the radius and the interosseous membrane of the forearm. The muscular part was 166.37 mm long. Then FPL passed into the tendinous part, which ended in two tendons. In the myotendinous junction, the width was 5.87 mm and the thickness was 2.36 mm. The length from this point to the point of division was 92.24 mm. At the point of separation the FPL was 7.10 mm wide and 2.87 mm thick.

Regarding the division of the distal attachment of this muscle into two tendons, the first (more lateral) was 30.08 mm long and the second 30.42 mm. Both insertions were located on the distal phalanx of the thumb in the palmar surface of the hand.

The additional head was 95.72 mm long. It originated at the medial epicondyle; here, the width was 7.24 mm and the thickness was 2.33 mm. The insertion of the additional head was attached to the upper part of the first head. At this point, the width was 5.17 mm and the thickness 1.87 mm.

The proximal attachment of the unrecognized structure comprised six small heads. The first (length 15.28 mm), third (length 12.25 mm) and sixth (length 21.73 mm) were connected to the FPL. The second (length 15.01 mm), fourth (length 21.93 mm) and fifth (length 22.19 mm) originated on the interosseous membrane of the forearm, medial to the attachment of the FPL. All of these heads merged with each other and created a single thin band 73.44 mm long. The distal attachment of this unknown muscular structure was also connected to the FPL, and at the point of connection, it was 5.30 mm wide and 1.87 mm thick.

The muscle was measured with an electronic caliper (Mitutoyo Corporation, Kawasaki-shi, Kanagawa, Japan). Each measurement was repeated twice with an accuracy of up to 0.1 mm. No other anatomical variations were identified during the dissection.

The morphometric measurements of the FPL are given in Table 1, and the morphometric measurements of the unrecognized structure in Table 2.

**Table 1:** Morphometric measurements of the FPL

Standard part of the FPL		
Origin	Anterior surface of the radius and interosseous membrane	
Insertion	Distal phalanx of the thumb (both insertions)	
Length		
Muscular part (mm)	166.37	
Tendinous part (mm)	94.24 + 30.08 (first insertion)	90.24 + 30.42 (second insertion)
Width		
Myotendinous junction (mm)	5.87	
Point of division of two insertions (mm)	7.10	
Thickness		
Myotendinous junction (mm)	2.36	
Point of division of two insertions (mm)	2.87	

Additional head of the FPL		
Length	95.72 mm	
	Proximal attachment	Distal attachment
Place of attachment	Medial epicondyle	FPL
Width (mm)	7.24	5.17
Thickness (mm)	2.33	1.87

**Table 2:** Morphometric measurements of the unrecognized structure

Proximal attachment						
Number of heads	I	II	III	IV	V	VI
Origin	FPLM	Interosseous membrane	FPLM	Interosseous membrane	Interosseous membrane	FPLM
Length (mm)	15.28	15.01	12.25	21.93	22.19	21.73
Length of the common part (mm)	73.44					

Distal attachment						
Insertion	FPL					
Width (mm)	5.30					
Thickness (mm)	1.87					

## Discussion

The first interesting thing is a possible cause of an occurrence such an additional structure. It may be associated with both, process of evolution and ontogenesis. As an example, we want to mention about case report described by Yurasakpong et al. [32], who recently reported an unknown fibromuscular structure associated with a variant on the dorsum of the hand. They believed it was a remnant of the dorsometacarpales, a group of primitive extensors found in mammalian ancestors. Maybe, in our case a cause is similar. However, in the flexor compartment, there is no structure which disappears in the course of ontogenesis. Moreover, the FPL appeared only during human evolution. On the other hand, it could be correlated with the progression of dexterity and more precise hand movements, so maybe it is something like an adaptive change. For that reasons, it is difficult to define the confirmed cause of such a structure [7].

The morphological variants in the case presented were of three sorts: associated with the proximal attachment of the FPL, associated with the distal attachment of the FPL, and occurrence of an unrecognized structure.

As mentioned above, the proximal attachment was represented by two heads, one of which was an additional head. This variant is surprisingly common, though the available literature shows large discrepancies in its reported frequency. For example, Sembian et al. [29] found that the additional head of the FPL (AHFPL) was present in 0.5% of upper limbs, while Mangini [15] reported its prevalence as 73.68%. This wide divergence of results is puzzling. One explanation could be erroneous classification. The fusion between the FPL, its additional head and the surrounding superficial flexors could lead to misidentification in some studies and a lower reported frequency.

These inaccuracies were the basis for a meta-analysis by Roy et al. [28]. Their results showed that the pooled prevalence of the AHFPL in upper limbs was 44.2%. A very similar frequency (45%) was noted by Dellon and Mackinnon [6]. The reported incidence of an additional head was significantly higher than average in some studies in this meta-analysis; for example, 67% by Hemmady et al. [9] and 74% by Mangini [15]. The meta-analysis results [28] also showed that an additional head of the FPL in cadavers was marginally more common bilaterally (prevalence 52.1%) than unilaterally (prevalence 47.9%). These statistics were consistent with the findings of, for example, Caetano et al. [4], Hemmady et al. [9], Mangini [15], Dellon and Mackinnon [6], Oh et al. [19], and Al Quattan [1].

Roy et al. [28] also noticed that the AHFPL is more common among men (the frequency was 41.1%) than women (24.1%). This could be closely related to the misclassification mentioned above because all structures in upper female limbs are smaller than those in males, so the AHFPL could more readily elude identification [28].

It is worth mentioning that the case presented includes an additional head of which the distal attachment is located on the medial epicondyle. According to the meta-analysis results [28], this is the most common type of AHFPL (43.6%). There are also cases in which the additional head is attached to the coronoid process of the ulna (25.8%). Sometimes it has a dual origin on the medial epicondyle and coronoid process (16.1%) [28].

The additional head has great clinical significance. It is implicated in Anterior Interosseous Syndrome (AIS). When the AIN courses underneath the additional muscle belly it can be compressed, causing AIS [2]. This is analogous, for example to an accessory subscapularis muscle potentially causing quadrilateral space syndrome [33], or the tendency of an additional head of the coracobrachialis muscle to compress the median and musculocutaneous nerves [27].

The AIN is provides motor innervation to the FPL, PQ, and the radial half of the FDP. Patients with AIS usually report dull pain in the forearm [2, 30]. A characteristic symptom of this neuropathy is an inability to form an “O” with the index finger and thumb because the FPL and FDP are paralyzed. Flexion of the interphalangeal joint of the thumb and the distal interphalangeal joint of the index finger is also impaired. Carpal tunnel syndrome causes similar symptoms, but it also causes numbness, paresthesia, and loss of sensation, which help to differentiate it [5].

AIS is closely associated with an AHFLP, as confirmed by Gunnal et al. [8]. They showed that in 90.21% of cases the AIN was located posterior to the additional head, predisposing to entrapment. Its location was anterior to the AHFPL in only 9.78%. AIN compression can also be called Kiloh–Nevin syndrome [11]. AIS can be complete or incomplete. Complete AIS occurs when the whole nerve is compressed by the accessory head and movements due to the FPL, FDP (index and middle fingers), and PQ muscles are impaired. Incomplete AIS occurs when only the medial branch of the AIN (innervating the FPL) passes underneath the additional belly [8].

The next part of our case is the morphological variation of the distal attachment of the FPL. The tendinous insertion was bifurcated. However, these two tendons had the same point of attachment (distal phalanx of thumb). We hypothesize that such an arrangement could have clinical significance. Division of the tendinous part of the FPL can indicate reduced strength but also the possibility of more complex movements [34]. The mechanical properties of any tendon will determine its tendency to strength. If material properties are held constant, a short tendon with a large cross-sectional area will tend to be stiffer than a few thin tendons [16]. Evolutionary analysis showed that the FPL only appeared during human evolution and is present in modern humans [7]. It can therefore be correlated with the progression of dexterity and more precise hand movements in humans than in previous evolutionary clades [34]. We hypothesize that the occurrence of two tendinous insertions increases the range of more precise movements.

The second advantage of this morphological variation is protection against tears in all tendinous part during repetitive movements or as a result of mechanical injury [13]. Flexor tendon lacerations are usually caused by injury by a knife, shard of glass, or sharp metal [7]. The FPL is the only muscle responsible for flexing the thumb at the distal interphalangeal joint, so when the tendinous parts are torn, this movement is abolished [7]. In our case, the two-tendon distal attachment allows muscle function to be maintained, though it is probably weakened. As mentioned above, the FPL is also responsible for other movements, but there it works synergistically with other muscles, so if there are tears there is still movement, albeit possibly weakened. Summing up these two paragraphs, the bifurcated tendinous part of the FPL could reflect an evolutionary adaptation that increases the range of more detailed movements and protects against tears and their effects.

The last but not least part of our case report is the unrecognized structure. Regarding its clinical significance, we considered two things: its potential function, and the possibility of compression of other structures. The proximal attachment of this structure comprised six distinct heads, three of which were connected to the muscular part of the FPL and three to the interosseous membrane of the forearm. We hypothesize that the potential functions of this muscle entail a complex mechanism. First, FPL tension could be transferred to this unknown structure by the three heads attached to it, potentially causing the additional organ to contract. Second, the three heads attached to the interosseous membrane could transfer shrinkage and contribute to tension of the interosseous membrane. This fibrous tissue provides stabilization for the forearm [18], so this too is a potential function for the unrecognized structure. The interosseous membrane is located between the radius and ulna and protects against excessive relative displacement of these two bones [18]. However, we think our additional structure is too small to influence this significantly.

Its distal attachment was inserted into the tendinous part of the FPL; this could also have functional implications. The force of the FPL muscle’s contraction could possibly be distributed over two muscles, the FPL and our unrecognized structure. This force is then transferred via the additional organ to the tendinous part of the FPL, and thus to the distal phalanx of the thumb. Such a process could help to protect against FPL fibrosis.

Additional structures typically compress nerves, arteries or other muscles. For example, an accessory subscapularis muscle can cause quadrilateral space syndrome [33]. A very rare variant of the coracobrachialis longus muscle can entrap the musculocutaneous, media, and ulnar nerves [23]. Compression is therefore another possible clinical significance of this unknown structure to be considered. However, its placement should not predispose to such a pathology.

In summary, the present case has clinical significance. Since the morphological variations can be divided into three sorts, the role of this unknown structure could be very diverse, ranging from potential compression to prevention of tendon rupture. As mentioned above, although the additional head of the FPL is a frequent variant, the unknown structure described here has not been reported in available literature.

### Conclusion

The flexor pollicis longus is characterized by high morphological variability. Research into variability in this region is essential for a proper understanding of the functioning of the hand and forearm. Further studies are also needed to understand the embryonic and evolutionary origin of this unrecognized structure.
